# Planning and delivery of intensity modulated bolus electron conformal therapy

**DOI:** 10.1002/acm2.13386

**Published:** 2021-09-24

**Authors:** Elizabeth N. Hilliard, Robert L. Carver, Erin L. Chambers, James A. Kavanaugh, Kevin J. Erhart, Andrew S. McGuffey, Kenneth R. Hogstrom

**Affiliations:** ^1^ Department of Physics and Astronomy Louisiana State University Baton Rouge Louisiana USA; ^2^ Mary Bird Perkins Cancer Center Baton Rouge Louisiana USA; ^3^ Department of Radiation Oncology Washington University School of Medicine Saint Louis Missouri USA; ^4^ .decimal, LLC Sanford Florida USA

**Keywords:** bolus electron conformal therapy, electron beams, intensity modulation

## Abstract

**Purpose:**

Bolus electron conformal therapy (BECT) is a clinically useful, well‐documented, and available technology. The addition of intensity modulation (IM) to BECT reduces volumes of high dose and dose spread in the planning target volume (PTV). This paper demonstrates new techniques for a process that should be suitable for planning and delivering IM‐BECT using passive radiotherapy intensity modulation for electrons (PRIME) devices.

**Methods:**

The IM‐BECT planning and delivery process is an addition to the BECT process that includes intensity modulator design, fabrication, and quality assurance. The intensity modulator (PRIME device) is a hexagonal matrix of small island blocks (tungsten pins of varying diameter) placed inside the patient beam‐defining collimator (cutout). Its design process determines a desirable intensity‐modulated electron beam during the planning process, then determines the island block configuration to deliver that intensity distribution (segmentation). The intensity modulator is fabricated and quality assurance performed at the factory (.decimal, LLC, Sanford, FL). Clinical quality assurance consists of measuring a fluence distribution in a plane perpendicular to the beam in a water or water‐equivalent phantom. This IM‐BECT process is described and demonstrated for two sites, postmastectomy chest wall and temple. Dose plans, intensity distributions, fabricated intensity modulators, and quality assurance results are presented.

**Results:**

IM‐BECT plans showed improved D_90‐10_ over BECT plans, 6.4% versus 7.3% and 8.4% versus 11.0% for the postmastectomy chest wall and temple, respectively. Their intensity modulators utilized 61 (single diameter) and 246 (five diameters) tungsten pins, respectively. Dose comparisons for clinical quality assurance showed that for doses greater than 10%, measured agreed with calculated dose within 3% or 0.3 cm distance‐to‐agreement (DTA) for 99.9% and 100% of points, respectively.

**Conclusion:**

These results demonstrated the feasibility of translating IM‐BECT to the clinic using the techniques presented for treatment planning, intensity modulator design and fabrication, and quality assurance processes.

## 1 INTRODUCTION

To date three types of electron conformal therapy (ECT) have been studied, bolus ECT (BECT), segmented‐field ECT, and modulated electron radiation therapy (MERT).^1^ Each has advantages and disadvantages compared with the others; however, only BECT technology is widely available in today's clinic. In BECT, a variable thickness bolus abutting the patient surface is used to modulate laterally the therapeutic range, for example, R_90_, so that the 90% dose surface conforms to (circumscribes) the patient planning target volume (PTV). BECT offers the potential for lower whole body dose, reducing secondary cancer risks, and equal or lower dose to nearby critical structures, reducing normal tissue complications, as compared to that of intensity modulated x‐ray therapy (IMXT).

Historically, electrons have been an important modality for (1) the treatment of skin, lip, head, and neck tumors, (2) boost doses to superficial lymph nodes, and (3) postmastectomy chest wall irradiation.^2^, ^3^, ^4^, ^5^ Since the onset of 3D treatment planning, BECT has been shown useful for posterior chest wall^6^, ^7^, ^8^; postmastectomy chest wall^7^, ^9^, ^10^, ^11^; ear, parotid, and buccal mucosa^7^, ^12^; nose^13^; and extremities (hand and foot).^8^ As a result, BECT is currently available from two companies that provide bolus design software, which integrate with one's treatment planning system,^8^, ^14^ and bolus fabrication methods (milled or printed).^6^, ^8^

The typical BECT treatment and delivery process consists of patient immobilization, CT scanning, PTV and normal tissue delineation, beam design, bolus design, dose calculation, bolus fabrication, and quality assurance.^6^ Bolus design is typically an iterative optimization process.^8^, ^14^ Physical quality assurance following fabrication can be made by bolus thickness measurement at multiple off‐axis locations. Clinical, dosimetric quality assurance can be made using a repeat CT scan from a CT simulator or pre‐treatment cone beam CT to perform a patient dose calculation with bolus in place.^6^, ^13^

Clinical experience has demonstrated that in some cases, the upstream bolus surface is sufficiently irregular to cause undesirable dose heterogeneities in the PTV, that is, volumes of increased dose (hot spots) and decreased dose (cold spots). However, Kudchadker et al.^7^ showed that the introduction of modest intensity modulation (70%–100%) across the beam, followed by moderate redesign of the bolus, can significantly reduce PTV dose heterogeneity while maintaining a dose distribution conformal to the PTV. Hence, intensity modulated BECT, that is, IM‐BECT, can conform the therapeutic dose surface (e.g., 90%) to the distal PTV surface, while producing a reasonably uniform dose in the PTV (≈10%–15% dose spread).

Recently, Hogstrom et al.^15^ developed a simple, potentially economical method for design and fabrication of a passive radiotherapy intensity modulator for electrons (PRIME) device,^16^ analogous to the utilization of x‐ray compensators for IMXT prior to the widespread availability of x‐ray multi‐leaf collimators (MLCs).^17^, ^18^ The objective of this paper is to describe a process that should be suitable for planning and delivery of IM‐BECT. The process, detailed and demonstrated for two patient sites, includes adding techniques for design, fabrication, and quality assurance of the intensity modulator^19^, ^20^ to the current BECT process.^6^

## 2 METHODS

Methods for planning and delivering IM‐BECT are demonstrated using data from two patients previously treated using BECT. For each patient, treatment planning was done using research versions of a BECT bolus design system in which the ability to utilize intensity modulators was added. The intensity modulators (PRIME devices) resulting from the treatment plans were fabricated, then the process of clinical quality assurance of the fabricated intensity modulators was demonstrated by comparing dose calculations with measurements beneath the intensity modulators in a water phantom. Planning and delivery techniques, that is, bolus and intensity modulator design, patient dose calculation, fabrication of intensity modulator, and quality assurance, are presented.

### 2.1 Patient data

**FIGURE 1:**
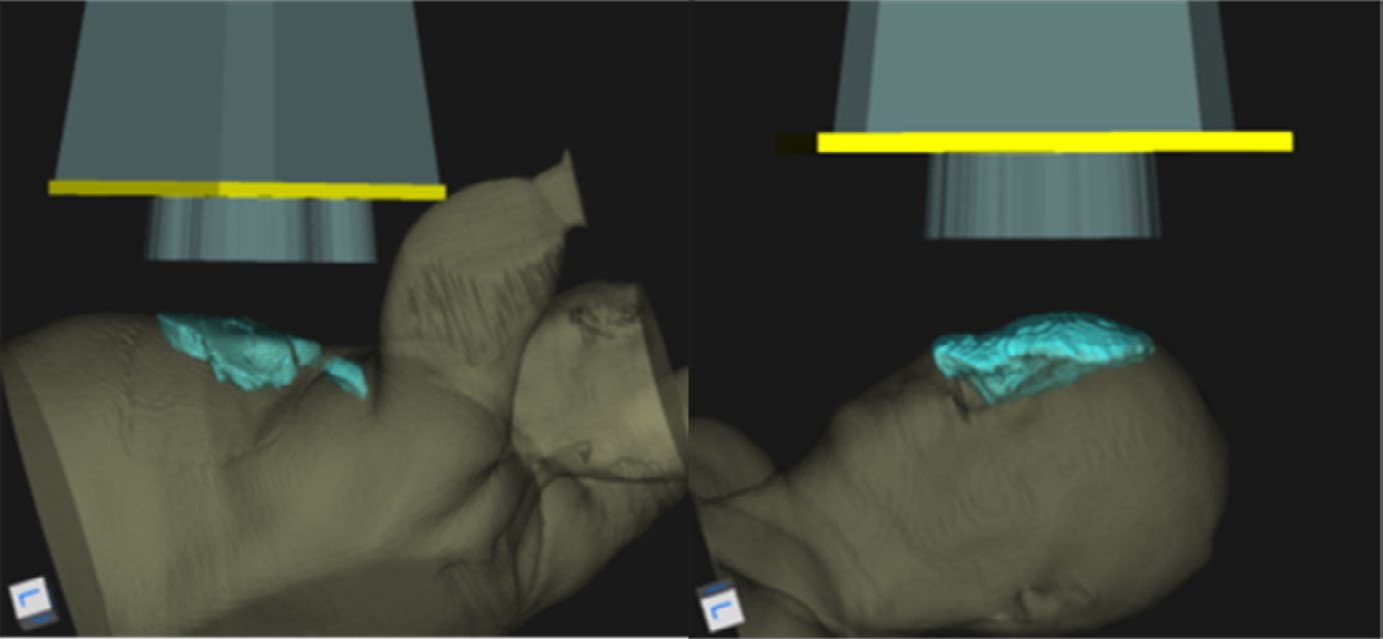
3D rendering of the postmastectomy chest wall (left) and temple (right) patient data used in current demonstration. The renderings show the skin surface, planning target volume (aqua), electron collimating insert (yellow), and electron beam (gray)

Two anonymized patient CT data sets (HIPAA compliant) were selected for two sites, postmastectomy chest wall and temple. Each patient had been treated previously with BECT. Chest wall and head and neck patients were selected because such patients have shown moderately and significantly improved PTV dose homogeneity when using IM‐BECT as opposed to BECT (Kudchadker et al.,^7^ Doiron^21^). This study used the planning target volumes (PTVs), normal tissues, and structures previously contoured by radiation oncologists and medical dosimetrists (*cf*. Figure 1).

### 2.2 Treatment planning

The patient plans for this study were developed using the Pinnacle^3^ treatment planning system (Philips) and research versions of the p.d bolus design system^22^ (.decimal, LLC). The beam geometry (gantry, couch, and collimator angles; source to surface distance, SSD; and table and isocenter locations), block aperture, and beam energy for each patient's previous BECT treatment, and the PTV outlines, structure outlines, and CT data for each patient were inputted from Pinnacle^3^ into the p.d software using DICOM transfer. For both patients the selected beam energy was 16 MeV (R_90_ = 5.0 cm).

The p.d software system designed the bolus and intensity modulator and calculated the dose distribution for each patient IM‐BECT plan. This process can be done in approximately 2 hours by a treatment planner competent with the IM‐BECT planning process and p.d software. Details of this treatment planning process and how it differs from a BECT treatment planning process are presented as follows.

#### 2.2.1 Initial bolus design

For each patient, a conformal bolus was designed using the clinical version (v5.1) of p.d.^22^ The bolus was designed such that the 90% dose surface conformed to (circumscribed) the distal PTV surface. The design process used a sequence of operators that created, modified, and extended the bolus. The Create (physical depth), Isodose Shift, Smooth, and Height Extension operators were those of Low et al.,^14^ and the specified shift operator shaved or added a specified uniform thickness to the bolus. The operators were used in sequences which best achieved the treatment planning goals, namely that the 90% isodose surface circumscribed the PTV. The Truncate operator removed upstream bolus surface outside the field to reduce bolus weight, having no impact on the dose distribution. The sequences of operators used to design the initial patient bolus for each patient are listed in Table 1.

**TABLE 1:** Sequence of p.d operators (parameters in parenthesis) used to design a bolus and intensity modulator for each patient^22^

Post‐mastectomy chest wall patient	Temple patient
Create (90%, 0.7 cm) Smooth (2,1) Isodose Shift (0.5 cm) Smooth (2,1) Isodose Shift (0.5 cm) Smooth (2,1) Truncate Specified Shift (−0.1 cm BECT, −0.3 cm IM‐BECT) **Intensity Modulation**	Create (90%, 0.7 cm) Smooth (2,1) Isodose Shift (0.5 cm) Smooth (2,1) Isodose Shift (0.5 cm) Smooth (2,1) Truncate Specified Shift (−0.2 cm BECT, −0.4 cm IM‐BECT) **Intensity Modulation**

The resulting intensity modulators were fabricated for QA measurements. The BECT operator sequence (un‐bolded steps) was followed by the intensity modulation sequence (bolded step). The Create operator input parameters are the percent dose (90%) being conformed to the distal PTV and the distance inside the PTV edge (0.7 cm) in which the bolus is created based on physical depth; the Smooth operator input parameters are a multiplier (2) in the exponent of a Gaussian function and a multiplier (1) of a 1.5‐cm radial distance over which the smoothing of the bolus height occurs; the Isodose Shift operator input parameter is the distance inside the PTV edge (0.5 cm) in which the bolus thickness is increased or decreased to shift the depth of the 90% dose surface within the patient; and the Specified Shift operator input parameter (−0.1 or −0.2 cm) is the thickness subtracted from the entire proximal bolus surface. Truncate minimizes the bolus mass by reducing the flat, upstream bolus surface outside the treatment field, having no impact on the dose distribution.

#### 2.2.2 Initial intensity modulator design

After completing the initial bolus design, the intensity modulation operator was applied to create greater dose homogeneity within the patient by reducing dose greater than 100% (hot spots, as dose typically is prescribed to 90%). These IM‐BECT treatment plans were created in a research version of p.d, which included an additional intensity modulation operator, as described by Hilliard.^20^ The intensity modulation operator requires the user to choose a limit to the maximum and minimum intensity reduction factors (IRFs). The program then designs an intensity map (IRF versus off‐axis position) based on the dose distribution resulting from the initial bolus design.

Because of a small interdependence of the bolus and intensity modulator,^7^ to achieve better patient plans the bolus requires reoptimization following application of the intensity modulation operator. The research version of p.d used for the initial intensity modulator design and fabrication did not allow for bolus reoptimization, but was sufficient for early bolus fabrication and QA measurements. A subsequent research version of p.d, which included bolus reoptimization, was used to generate the final dose distributions used to evaluate patient plans. Although the two research versions differed slightly, differences in their designed intensity modulators were clinically insignificant (<1%).

The sequences of operators used to design the fabricated intensity modulators are shown in Table 1. The sequences of operators used for the final patient plans, which includes bolus reoptimization are shown Table 2. Bolus reoptimization was not required for the temple patient.

**TABLE 2:** Sequences of p.d operators (parameters in parenthesis) used for comparing BECT and IM‐BECT patient plans. BECT plans used the operator sequence (non‐bolded steps); IM‐BECT plans used the same BECT sequence plus the intensity modulation and bolus reoptimization sequence (bolded steps)

Post‐mastectomy chest wall patient	Temple patient
Create (90%, 0.5 cm) Smooth (2,1) Isodose Shift (0.5 cm) Smooth (2,1) Isodose Shift (0.5 cm) Smooth (2,1) Truncate Specified Shift (−0.1 cm) **Intensity Modulation** **Isodose Shift (0.5 cm)** **Smooth (2,1)** **Specified Shift (−0.1 cm)**	Create (90%, 0.7 cm) Smooth (2,1) Isodose Shift (0.5 cm) Smooth (2,1) Isodose Shift (0.5 cm) Smooth (2,1) Truncate Specified Shift (−0.2 cm) **Intensity Modulation**

##### Modification of pencil beam weights

The intensity modulation operator modifies the weight of each pencil beam by looking at the maximum dose along each ray line from the beam's virtual electron source (assumed 100 cm from isocenter) through the center of each pencil beam, which falls a specified margin (0.5 cm) inside the edge of the PTV. The pencil beam weight along the *i*,*j* fan line $\left(w_{i,j}\right)$ is modified so that

(1a) $${\left(w_{i,j}\right)}_{\mathrm{new}}={\left(w_{i,j}\right)}_{\mathrm{old}}*IRF,$$

where

(1b) $$IRF=1.000\;\;\;\mathrm{for}\;D_{\max}\left(i,j\right)<100\%,$$

(1c) $$IRF=100\%/D_{\max}\left(i,j\right)\;\;\;\mathrm{for}\;{100\%\leq D}_{\max}\left(i,j\right)\leq 80^{‐1}\%,$$

(1d) $$IRF=0.800\;\;\;\mathrm{for}\;D_{\max}\left(i,j\right)>{80\%}^{‐1},$$

where $D_{\max}\left(i,j\right)$ is the maximum percent dose along the *(i*, *j)* pencil beam ray‐line, and the intensity reduction factor (*IRF*) was limited to the range from 0.8 to 1.0. Pencil beam weights for pencil beams with ray‐lines outside of the specified margin were determined using the same method Low et al.^14^ used for bolus height extension.

##### Segmentation of pencil beam weights into island blocks

**FIGURE 2:**
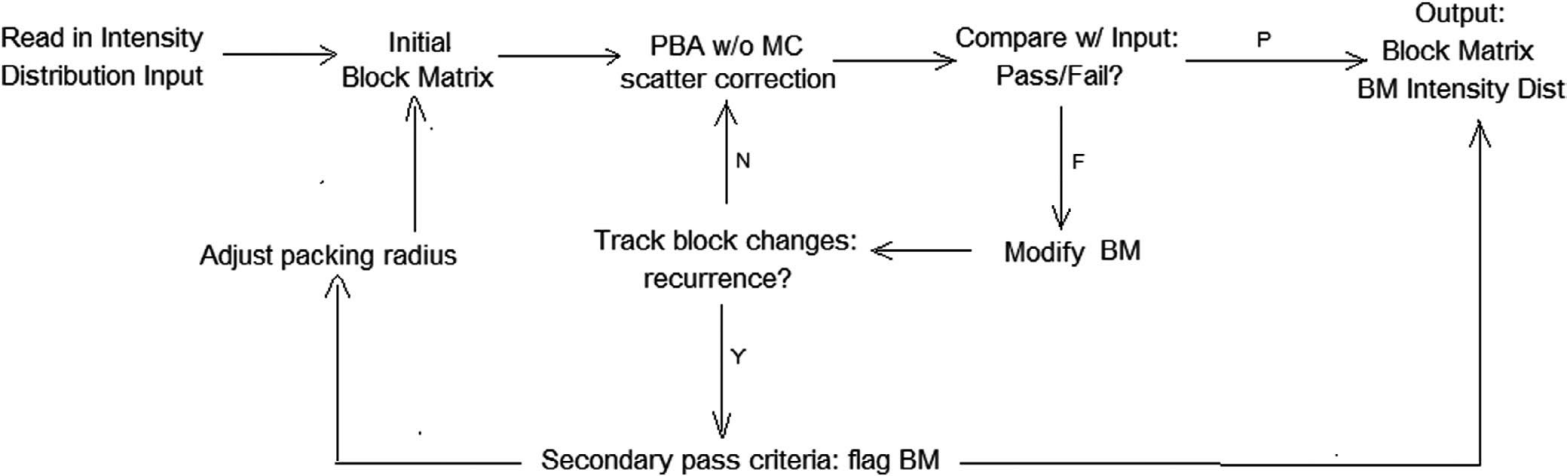
Workflow for the electron intensity modulator generator software. An initial block matrix using equation (2) is generated and tested using pre‐established criteria. Points of failure are improved iteratively until criteria are met or minimized. Abbreviations: PBA is pencil beam algorithm; BM is Block Matrix

Similar to an x‐ray intensity modulated field being segmented into an MLC leaf sequence, the intensity map of the “new” beam weights was segmented into an island block pattern that closely achieved that intensity map. The segmentation process was based on an algorithm developed by Chambers.^19^ It takes the initial island block pattern, determined using the method described below, then calculates the underlying intensity pattern using a pencil beam algorithm.^23^ After comparing the calculated with the desired intensity pattern, island block diameters are modified. This process, which iterates until results meet an optimization criteria, is shown schematically in Figure 2.

In the current study, only the initial step in the chambers optimization algorithm was used. Island blocks were placed on a 0.6‐cm hexagonal grid (specified at 93.5 cm source to collimator distance). For each grid point on the hexagonal grid the algorithm first calculated the island block diameter d based on fraction of area blocked using^15^

(2) $$d\left(r,\;IRF\right)=r{\left[\frac{2\sqrt{3}}{\pi }\left(1‐IRF\right)\right]}^{1\;/\;2},$$

where r is the distance separating the hexagonally packed circular island blocks of diameter *d*, and IRF is the intensity reduction factor, which equals the ratio of the desired underlying intensity to that in the absence of the intensity modulator, that is, $\left(w_{i,j}\right)$. Then, the algorithm selected an island block diameter closest from a set of available tungsten island block diameters (*cf*. Table 3). The resulting island block patterns were sent to the p.d bolus design system for the final dose calculation and to .decimal for fabrication of the intensity modulator.

**TABLE 3:** Available tungsten island block diameters *d* and corresponding IRF values when used with 0.6 cm hexagonal spacing r

*d* (cm)	0.158	0.223	0.273	0.315	0.352	0.386	0.417	0.473
*IRF* (%)	93.7	87.5	81.2	75.0	68.8	62.5	56.2	43.6

#### 2.2.3 Dose calculation using the pencil beam redefinition algorithm (PBRA)

For its electron beam dose calculations p.d bolus design software utilizes the pencil beam redefinition algorithm (PBRA), developed originally by Shiu and Hogstrom^24^ and improved by Boyd et al.^25^ to include a polyenergetic, incident electron beam. Dose at each point (*x*, *y*, *z*) is the sum of the electron beam component ($D^{e}$) and the background x‐ray dose component ($D^{X}$), that is,

(3) $$D\left(x,y,z\right)=D^{e}\left(x,y,z\right)+D^{X}\left(x,y,z\right),$$

where the x‐ray dose calculation uses an empirical, data based model described by Shiu.^26^ The electron dose calculation transports the phase space of the electron pencil beams at z to pencil beams at *z* + *Δz* modelling collisional energy loss, multiple Coulomb scattering, and beam divergence.

The PBRA was extensively validated by Boyd et al.^27^ using a measured data set by Boyd et al.^28^ for a number of patient‐like geometries and using Monte Carlo calculations for multiple patient sites.^29^ The PBRA was also validated in anthropomorphic phantoms for use with BECT by Carver et al.^30^

##### Changes to the PBRA for intensity modulators

For dose calculations with the passive intensity modulators containing island blocks, used in this study, three modifications were made to the PBRA: (1) reducing the energy of the beam to decrease R_90_ by 0.1 cm, which accounts for energy loss in the 1.27‐cm thick machinable foam (ρ = 0.096 g cm^−3^) containing the island blocks, (2) increasing the initial angular spread $\sigma_{\theta x}$ by 50% to account for additional scatter in the machinable foam, and (3) modelling the effect of the island blocks removing electrons from the beam. Details of the former two are described by Hilliard.^20^

The third modification of the PBRA code accounted for the reduction in electron fluence reaching the bolus surface due to the island blocks embedded in the intensity modulator's machinable foam. It was assumed that all electrons incident on the top surface of the island blocks are removed from the beam. This required modifying only the first step of the PBRA, which transports electron fluence from the plane of the collimator to the surface of the bolus (or patient). Each circular island block was modelled as a square pencil beam of equal area, whose electrons were transported to the surface of the bolus (or patient) with negative fluence.

**FIGURE 3:**
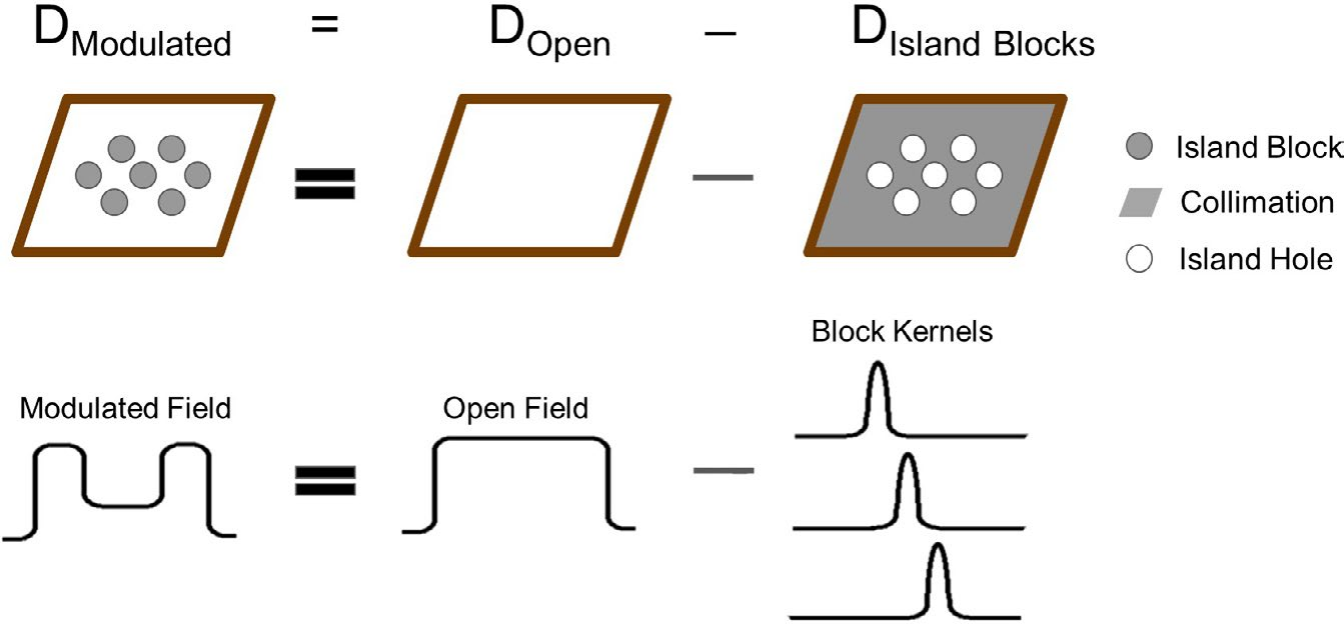
Schematic drawing showing how the PBRA code incorporated island blocks into the dose calculation for intensity modulated electron beams using the passive intensity modulators with island blocks

This concept, illustrated in Figure 3, determines the fluence at the starting pixel for the first pencil beam along each (i,j) ray line, which is given by

(4) $$\begin{array}{cc} \phi_{i,j}^{\text{intensity modulated beam}} & =\phi_{i,j}^{\text{beam without island blocks}}\\ & \;‐\sum_{l=1}^{\#\;\text{blocks}}\phi_{i,j}^{l} \end{array},$$

where $\phi_{i,j}^{l}$ is the fluence that would be contributed by electrons striking the *l^th^* block in its absence to the (i,j) pencil beams. This approximation ignored (1) electrons scattered into or out of the sides of the island blocks, that is, assumes perfect collimation, and (2) any changes to the bremsstrahlung dose caused by the island blocks.

### 2.3 Intensity modulator fabrication

Once the intensity modulators (PRIME devices) were designed, they were fabricated by .decimal using the following procedure. First, a standard patient‐specific 1.48‐cm thick copper cutout was machined. Second, a 1.27‐cm thick machinable foam insert matching the shape of the copper cutout was machined, and holes with axes following diverging rays from the virtual source were drilled 0.9 cm deep with the appropriate diameter to match the designed island block at the locations on the hexagonal grid having island blocks. Third, the milled machinable foam block was press‐fitted into the copper cutout. Fourth, the appropriate diameter 0.6‐cm long tungsten pin was manually inserted into each hole, completing the device. The entire process took approximately 2 hours, although some manual steps could be automated in the future.

### 2.4 Intensity modulator quality assurance

IM‐BECT requires at least three patient‐specific devices: wax bolus, electron cutout, and intensity modulator (occasionally skin collimation, internal collimation, or eye blocks are also used). Wax bolus quality assurance (QA) is the same for IM‐BECT as for BECT, which presently, is typically achieved by factory measurements and clinical acquisition of either a simulation CT scan with bolus^1^, ^6^ or pre‐treatment cone‐beam CT scan.^13^ Alternatively, the clinical QA could be a CT scan of the bolus. This section focuses on quality assurance for the intensity modulator, which by its nature also provides quality assurance of the collimating cutout that contains the intensity modulator.

#### 2.4.1 Factory quality assurance

**FIGURE 4:**
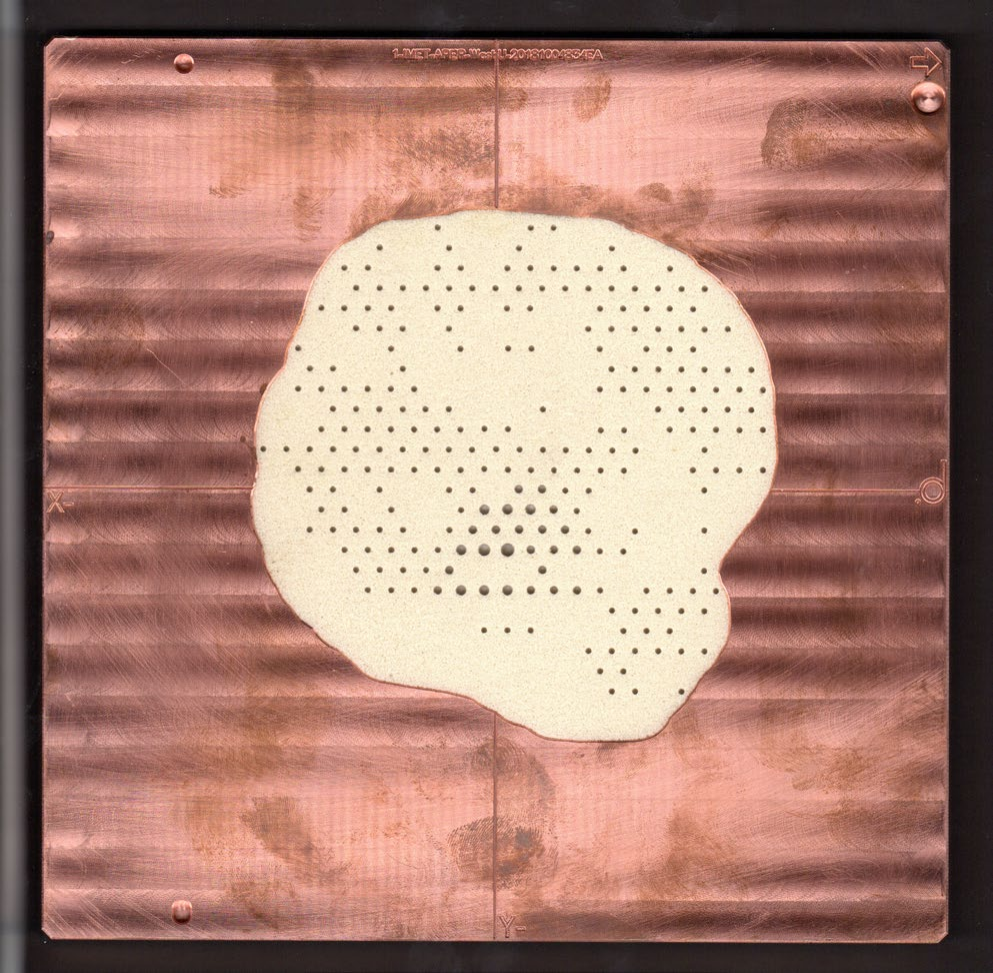
Optical scan of temple intensity modulator used for factory quality assurance. Shown is the custom copper cutout containing tungsten island blocks inserted into the machinable foam

The objective of factory quality assurance was to ensure that the correct diameter island blocks were inserted into the machinable foam substrate at the correct locations on the hexagonal grid. This was achieved by capturing an image of the intensity modulator (*cf*. Figure 4) using an optical scanner. A software program then used lines scored on the copper cutout that demarcate the beam orthogonal axes to determine planned locations of island blocks on the specified hexagonal grid. Second, the software validated the presence or absence of an island block. Third, the proper island block diameter was manually verified. Following factory quality assurance, the copper cutout‐intensity modulator was shipped to Mary Bird Perkins Cancer Center (MBPCC).

#### 2.4.2 Clinical quality assurance

The clinical quality assurance (QA) process mimics that used for x‐ray intensity modulation (generated by either a metal compensator or multi‐leaf collimator) in which the underlying intensity pattern is measured in a plane perpendicular to beam central axis. In the present study, measurements were made at 0.5 and 2.0‐cm depths in a water phantom at 100‐cm SSD using a scanning diode. These measurements were then compared with the PBRA calculated off‐axis dose distributions generated in p.d after replacing the patient and bolus with a water phantom. Our objective was to verify that the patient‐specific intensity modulator delivered a relative fluence (dose) distribution within 3% or 0.3 cm of that calculated by the PBRA.

##### Calculation of dose distributions downstream of patient intensity modulators

Relative dose (fluence) distributions were calculated using a research version of p.d. The dose was normalized such that 100% equaled the “given dose” without the intensity modulator. “Given dose” is the maximum central axis dose in water, *D*_max_ at R_100_, for the patient's effective field size (rectangular field with minimum area that circumscribes the irregular patient field), energy, and SSD. SSD was the distance from a point 100 cm upstream of isocenter to the bolus surface on central axis.

For comparison to measured dose distributions, the dose distributions were calculated in a water phantom with the patient intensity modulator (device cutout and island block pattern) for each patient. The dose was then transferred from p.d to the Pinnacle^3^ (v9.10 Philips) treatment planning system so that planar dose distributions could be exported to a laptop computer using MATLAB software (R2016, MathWorks). The planar dose ASCII files exported from Pinnacle^3^ contained a grid of calculated dose with pixel sizes of 0.1 cm.

##### Dose measurement downstream of patient intensity modulators

Off‐axis profiles were measured using an electron diode in a Blue Phantom^2^ (IBA Dosimetry) 3D scanning system; a CC13 ion chamber was used as the reference detector. The detectors were connected to a beam scanning common control unit (CCU), which contained two internal electrometers. The water phantom servo and CCU were controlled using the OmniPro‐Accept scanning software (v7, IBA Dosimetry). The water surface was set to 100‐cm SSD. Planar dose measurements were made at depths of 0.5 and 2.0 cm in water. Details of phantom setup are standard and have been reported by Hilliard.^20^

All measurements were performed on an Elekta Infinity accelerator, whose commissioned central‐axis depth doses were used by the PBRA. Measured doses were normalized in the identical manner as was the calculated dose with 100% equaling the “given dose”.

##### Methods for comparing calculated and measured dose distributions

The calculated and measured planar dose distributions were compared using isodose plots at the two measured depths (0.5 cm and 2.0 cm) for the intensity modulator of each of the two IM‐BECT treatment plans. Results at 2.0 cm are recommended for clinical QA and are presented here; results at 0.5 cm were similar and are reported by Hilliard.^20^ The percent dose difference and distance to agreement (DTA) between the calculated and measured distributions were calculated, where the DTA is defined as the distance from the point being examined and the closest point where the dose distributions have the same value. All points receiving at least 10% of the maximum dose were included in the percent dose difference/DTA analysis.

## 3 RESULTS

### 3.1 Patient treatment plans

**FIGURE 5:**
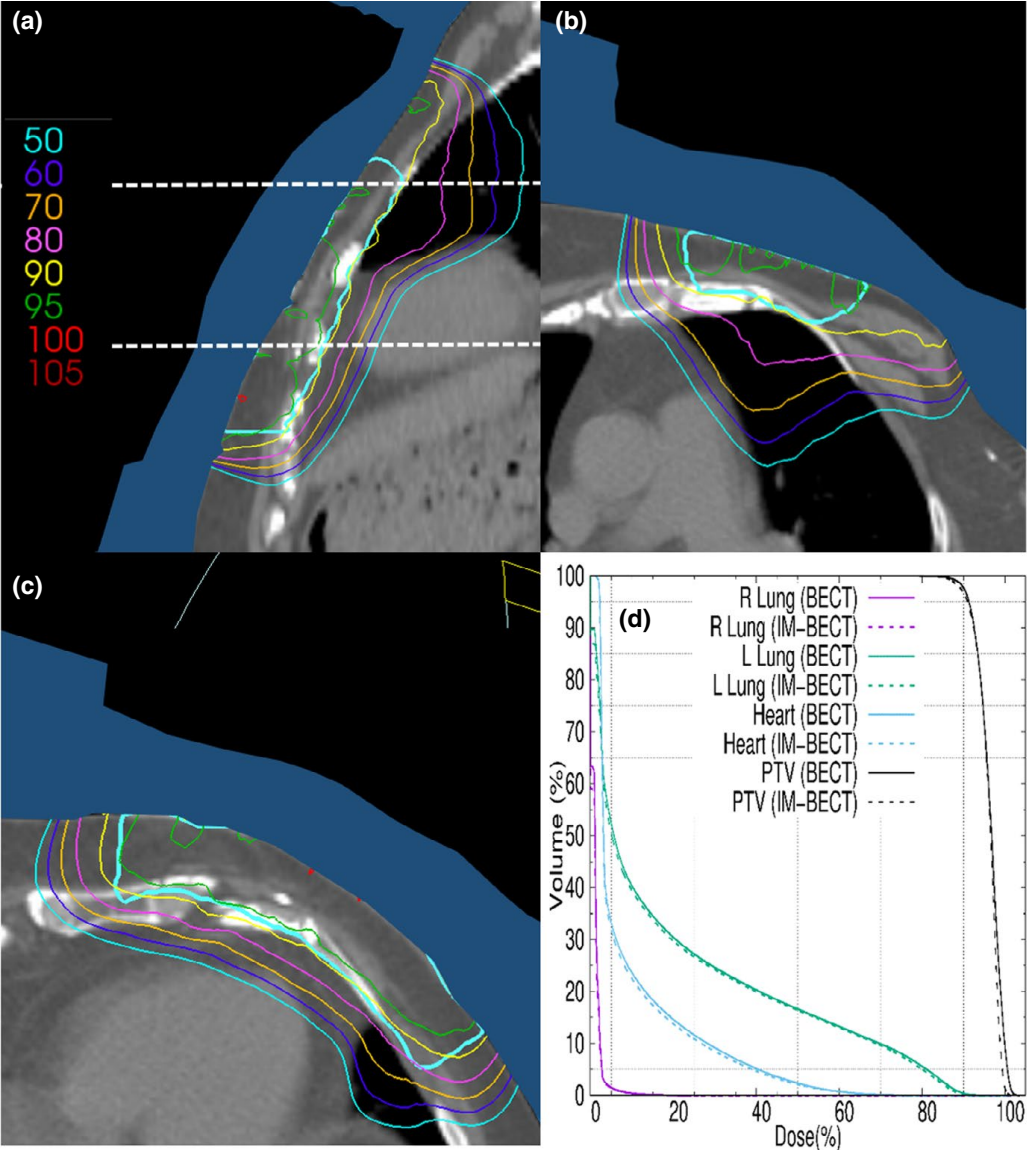
Chest wall patient CT planes showing the PTV (teal) and isodose lines (90% is yellow) for IM‐BECT treatment plan. The bolus is shaded blue. (a) Sagittal plane containing the central axis of the beam; dashed lines indicate positions of axial planes. Axial planes are (b) 3.5 cm superior (lung) and (c) 3.5 cm inferior (heart) to the central axis of the beam. (d) PTV, heart, right lung, and left lung DVHs for IM‐BECT and BECT treatment plans

The chest wall patient's IM‐BECT dose distribution is plotted for selected CT planes in Figure 5a–c. The sagittal plane demonstrates PTV coverage with respect to the heart and lung; the two transverse planes further demonstrate PTV coverage in planes through the heart and through the lungs. Figure 5d compares PTV, lung, and heart dose volume histograms (DVHs) for the IM‐BECT and BECT plans. DVHs were normalized such that the V_95_ for the PTVs match between the BECT and IM‐BECT plans. The IM‐BECT PTV DVH shows dose homogeneity improved from that of the BECT plan; the D_90‐10_ is reduced from 7.3% to 6.4%, and the maximum PTV dose is reduced from 103.6% to 101.4%. These are due to reducing the intensity inferiorly and laterally.

**FIGURE 6:**
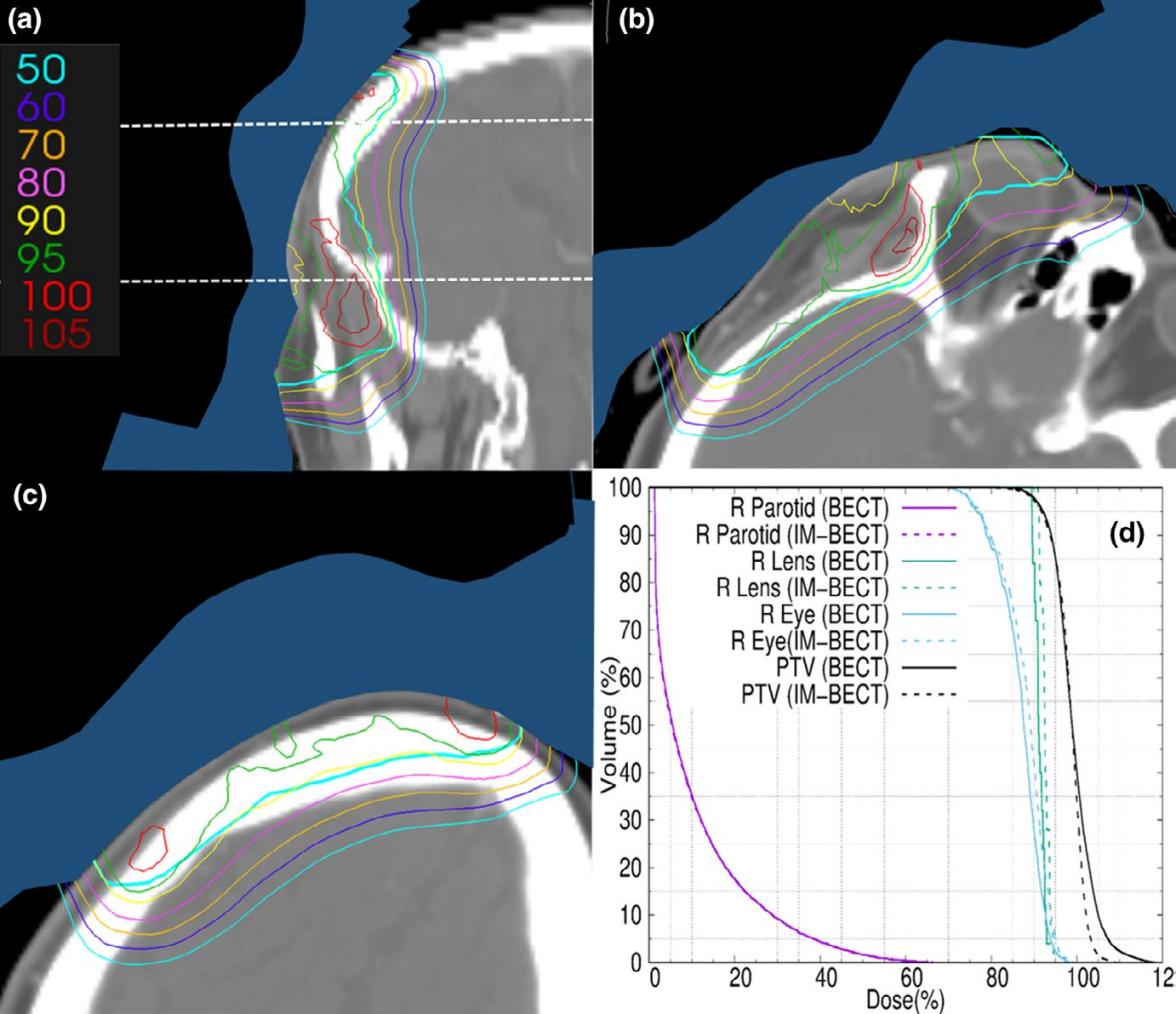
Temple patient CT planes showing the PTV (teal) and isodose lines (90% is yellow) for IM‐BECT treatment plan. The bolus is shaded blue. (a) Sagittal plane containing the central axis of the beam; dashed lines indicate positions of axial planes. Axial planes are (b) 3.0 cm inferior (R eye and brain) and (c) 3.0 cm superior (brain) to the central axis of the beam. (d) PTV, right eye, right lens, and right parotid DVHs for IM‐BECT and BECT treatment plans

The temple patient's IM‐BECT dose distribution is plotted for selected CT planes in Figure 6a–c. The sagittal plane demonstrates PTV coverage with respect to the temple and brain; the two transverse planes further demonstrate PTV coverage in planes through the right eye and brain. Figure 6d compares PTV DVHs for the IM‐BECT and BECT plans. DVHs were normalized such that the V_95_ for the PTVs match between the BECT and IM‐BECT plans. The IM‐BECT DVH shows dose homogeneity improved from that of the BECT plan; the D_90‐10_ is reduced from 11.0% to 8.4%, and the maximum PTV dose is reduced from 118.6% to 109.8%. These are due to reducing the intensity over the thinner regions of the bolus, which had underlying hot spots without intensity modulation.

### 3.2 Intensity modulator design and fabrication

**FIGURE 7:**
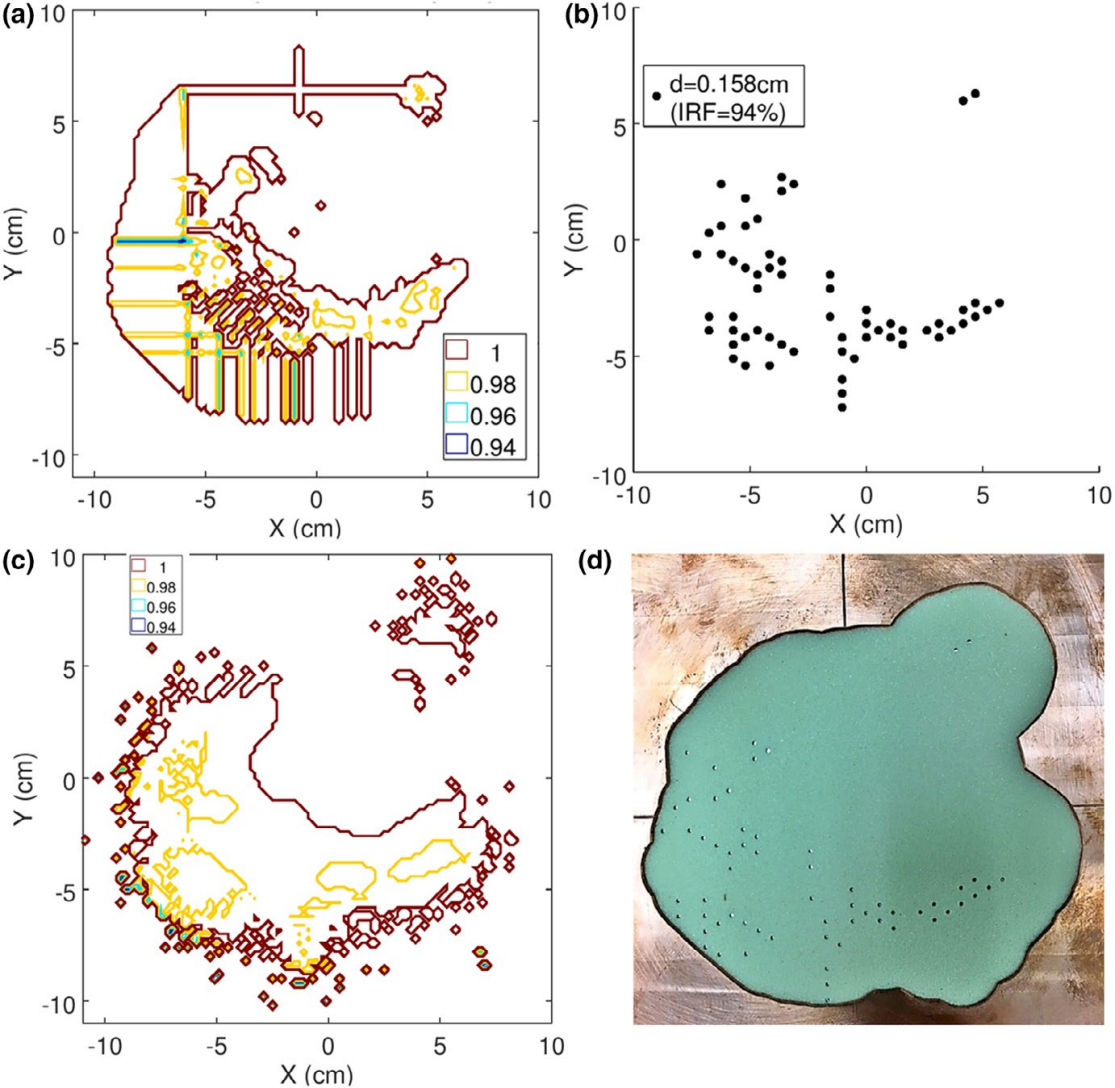
(a) Chest wall objective isointensity map as calculated by p.d (b) Pin pattern design resulting from segmentation of objective intensity map as described in Section 2.2.2. (c) PBA‐calculated intensity map based on pin pattern. (d) Photograph of intensity modulator fabricated for the chest wall site IM‐BECT treatment plan. (‐X is medial; ‐Y is inferior)

The isointensity distribution determined in the treatment planning process for the chest wall patient is mapped in Figure 7a. The range of intensity modulation was small in this case (0.94–1.00). It was mostly needed in the vicinity of the medial, inferior border of the field, due to angled incidence and shorter SSD of the patient there. This isointensity distribution was used to determine the island block distribution, which is shown in Figure 7b. The resulting isointensity distribution created by the island blocks is illustrated in Figure 7c by plotting the PBA‐calculated relative fluence distribution at a 0.5‐cm depth in a water phantom at 105 cm SSD. A photograph of the intensity modulator fabricated by .decimal is shown in Figure 7d. This intensity modulator required only 61 island blocks (all 0.158 cm diameter) placed at the specified locations on a 0.6‐cm hexagonal grid.

**FIGURE 8:**
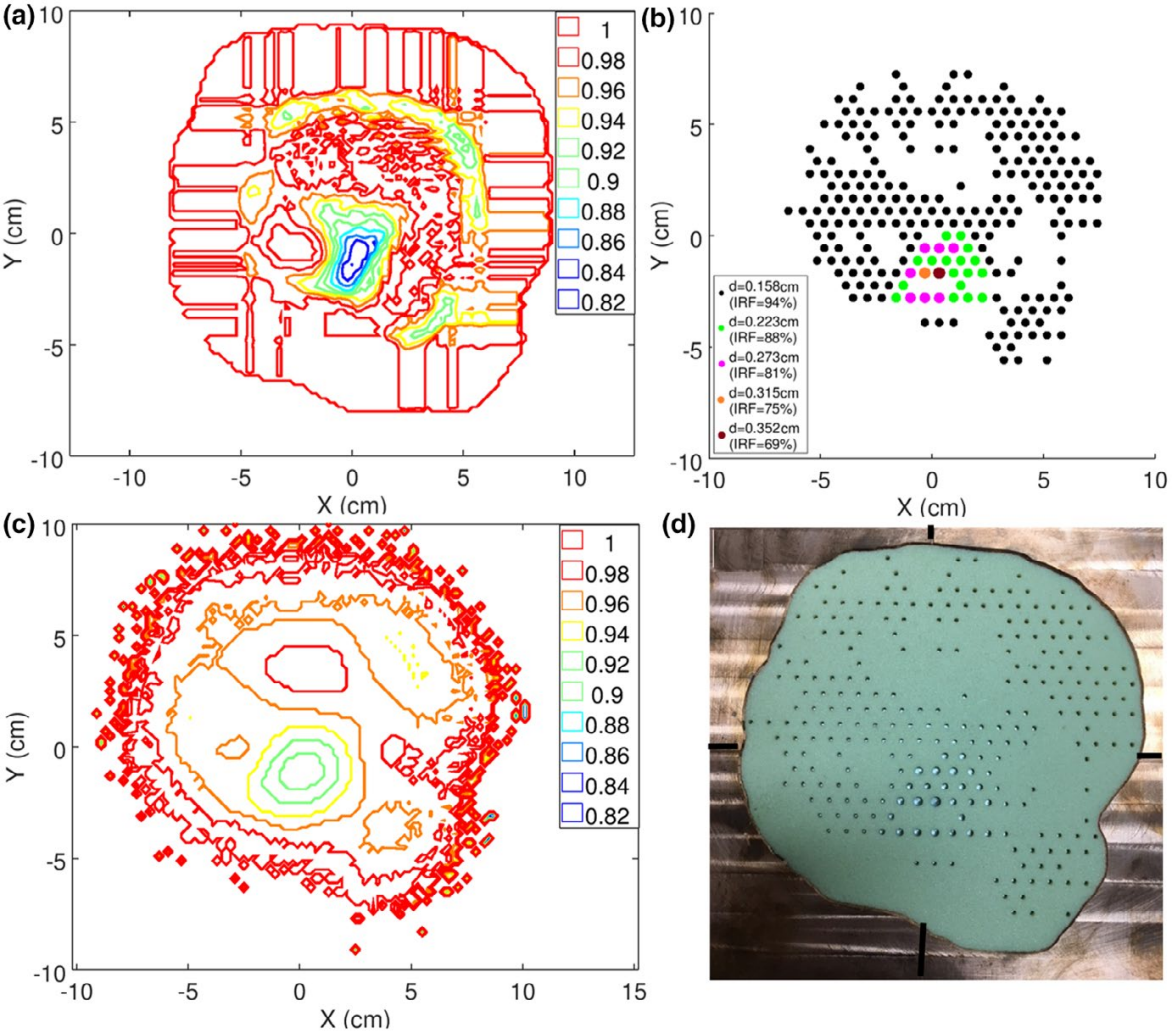
(a) Temple objective isointensity map as calculated by p.d (b) Pin pattern design resulting from segmentation of objective intensity map as described in Section 2.2.2. (c) PBA‐calculated intensity map based on pin pattern. (d) Photograph of intensity modulator fabricated for the temple IM‐BECT treatment plan. (‐X is medial/anterior; ‐Y is inferior)

Similarly, the isointensity distribution determined in the treatment planning process for the temple patient is mapped in Figure 8a. The range of intensity modulation was moderate in this case (0.82–1.00). It was mostly needed in the inferior portion of the anterolateral PTV region where the bolus was thinnest. Here, in‐scatter from the surrounding thicker portions of the bolus, which created considerable variability (gradients) of the bolus surface, resulted in hot spots. This isointensity distribution was used to determine the island block distribution, which is shown in Figure 8b. The resulting isointensity distribution created by the island blocks is illustrated in Figure 8c by plotting the PBA‐calculated relative fluence distribution at a 0.5‐cm depth in a water phantom at 105 cm SSD. A photograph of the intensity modulator fabricated by .decimal is shown in Figure 8d. This intensity modulator required 246 island blocks (1 at 0.352, 1 at 0.315, 7 at 0.273, 17 at 0.223, and 220 at 0.158 cm diameter) placed at the specified locations on a 0.6‐cm hexagonal grid.

### 3.3 Quality assurance of intensity modulator and bolus

Quality assurance of the intensity modulator at both the factory and clinical site occurred. Factory quality assurance used optical scanned images (similar to photographs in Figures 7d and 8d) to automatically validate pins were inserted at the desired locations on a hexagonal grid. Pin diameters were verified manually.

**FIGURE 9:**
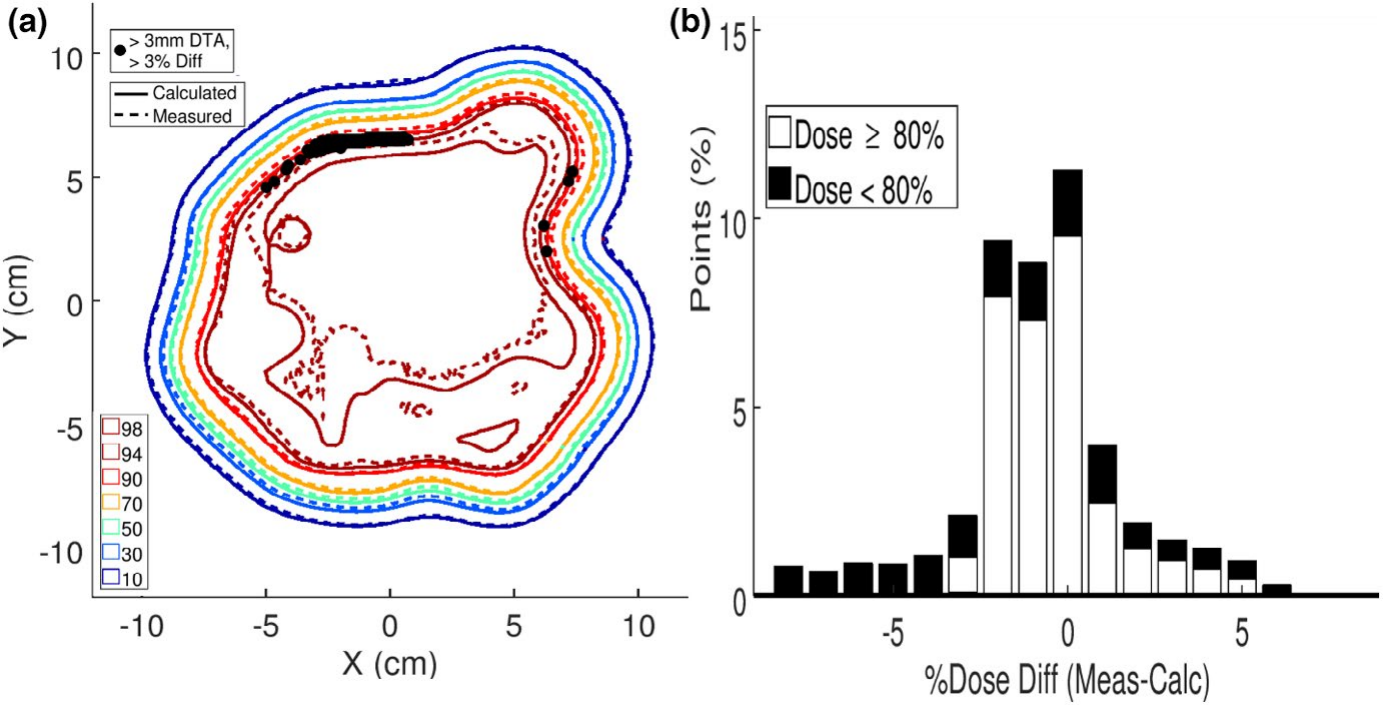
(a) For the chest wall patient, calculated (solid) and measured (dashed) isodose plots downstream of the intensity modulator at 2.0‐cm depth in water. Points not meeting the 3% or 3 mm criteria are highlighted in black (measured > calculated). (b) For the chest wall patient, histogram of the dose differences between the calculated and measured isodose data. Highlighted in black are the points receiving <80% of the given dose

**FIGURE 10:**
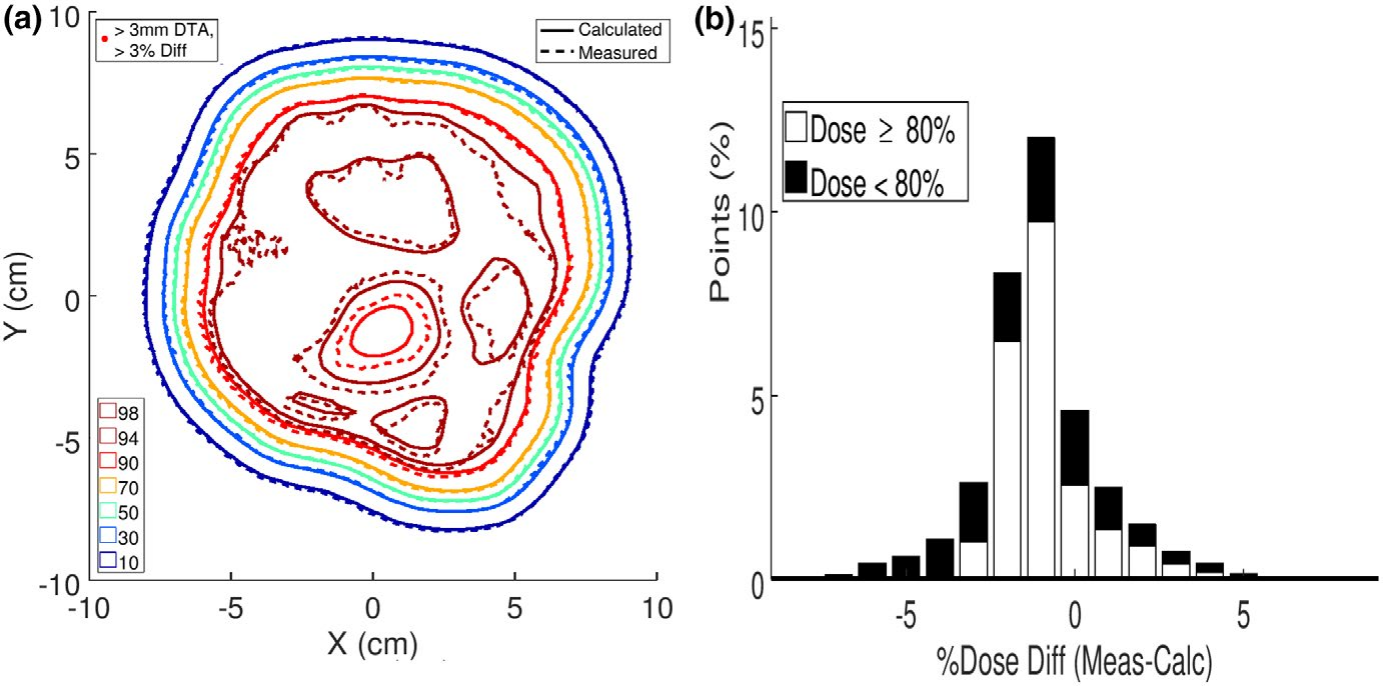
(a) For the temple patient, calculated (solid) and measured (dashed) isodose plots downstream of the intensity modulator at 2.0‐cm depth in water. All points met the 3% or 3 mm criteria. (b) For the temple patient, histogram of the dose differences between the calculated and measured isodose data. Highlighted in black are the points receiving <80% of the given dose

Clinical quality assurance was done by measuring the relative dose distribution at a 2.0‐cm depth in a water phantom underlying the intensity modulator. The measurement was compared with PBRA calculations from the planning system (patient and bolus replaced with water phantom). Isodose plots are compared for the chest wall and the temple patients in Figures 9a and 10a, respectively. Regions in which calculated dose exceeds 10%, measured dose differs by 3%, and distance to agreement (DTA) exceeds 0.3 cm are demarcated. Only small areas near the superior penumbra of the chest wall patient field exceeded that criteria, having a 99.9% pass rate; the temple intensity modulator had a 100% pass rate. Also, it should be appreciated that this QA test not only validates the intensity modulator, but also the electron cutout.

Another way of evaluating the dose comparisons is plotting dose difference histograms, which are plotted in Figures 9b and 10b for the chest wall and temple, respectively. These result show agreement within approximately (−3%, +5%,) and (−3%, +4%), respectively, for calculated dose greater than 80%.

## 4 DISCUSSION

### 4.1 Impact potential of IM‐BECT

In IM‐BECT the purpose of intensity modulation is to improve PTV dose homogeneity as compared to BECT, as first reported by Kudchadker et al.^7^ For this report, the two patient cases illustrating the IM‐BECT technique were selected based on their having been treated with BECT and having hot spots in their PTVs. Results showed IM‐BECT to improve dose homogeneity in the PTV by a modest amount; D_90‐10_ was reduced from 7.3% to 6.4% for the chest wall case and 11.0% to 8.4% for the temple case, somewhat less than the 14.9% to 9.2% for the buccal mucosa case of Kudchadker et al.^7^ Generally, electron beam therapy offers the potential to reduce secondary cancer control probability and dose to distal structures, the latter being enhanced by using BECT. IM‐BECT offers opportunity for improving BECT PTV dose homogeneity. Overall, the utility of IM‐BECT will depend upon how much normal tissue dose/complication probability and secondary cancer control probability might be reduced compared to current techniques. This suggests the need for patient studies to determine which sites might benefit most from IM‐BECT as compared to BECT, IMXT, and other current techniques.

The primary purpose of the current study was to expound on a process of using passive intensity modulators for IM‐BECT by further describing its planning, fabrication, and quality assurance techniques. As with any new technology, research and development is an ongoing process, and potential refinements of these individual clinical techniques are discussed below.

### 4.2 Reapplication of IM Operator in IM‐BECT treatment planning

Following application of the intensity modulation operator and bolus redesign, using an earlier research version of the p.d software (with intensity modulator operator, but without island block segmentation), Doiron^21^ showed benefit from one to two or more iterations of reapplying the intensity modulation operator and bolus redesign. The research version of the p.d software used in the present study included island block segmentation, but did not allow such further iterations. Hence, the IM‐BECT plans in the present study might not necessarily be optimal, but were suitable for illustrating IM‐BECT planning, fabrication, and quality assurance techniques. Future versions of IM‐BECT software will include such capability prior to clinical release. Also, clinical use might be further improved through research and development of methods for simultaneous optimization of bolus and intensity modulator design.

### 4.3 Quality assurance of intensity modulators

The factory QA process verified proper fabrication of the intensity modulator, that is, that island blocks are in the proper locations with the proper diameters. The locations were determined using computer software and an optical scanned image of the intensity modulator, and the diameters were verified manually; however, as the number of fabricated intensity modulators increases and prior to clinical release, software to determine island block diameters will be implemented.

Regarding the clinical QA process, clinical pass rates should be studied using a diode or ion chamber matrix, as such measurement techniques are more standard and practical than a beam scanning water phantom system. Also, a number of intensity modulators should be studied to better determine passing criteria and rates using current methods or gamma analysis and to investigate their dependence on minimum IRF and patient site.

### 4.4 Accuracy of PBRA dose calculation

Small differences between measured and calculated doses is expected due to the current PBRA calculation assuming perfect island block collimation, that is, scatter into and from the sides of the block has not been modeled. Preliminary data of Hilliard^20^ and more recent ongoing measurements showed PBRA calculations can underestimate measurements at 0.5 and 2.0 cm depths by as much as 5% in the energy range of 7–20 MeV for a large array of island blocks that create an IRF of 60%. Such extreme modulation is highly unlikely for IM‐BECT, hence this assumption had little impact on the accuracy of the PBRA calculated dose in the current study, as illustrated by the QA results. However, for other applications, this might not always be the case; therefore, scatter into and from the sides of the island blocks is being investigated further and in the future will be modeled in the PBRA.

### 4.5 Clinical Implementation

Clinical implementation of IM‐BECT is envisioned as an addition to the BECT process, which has been clinically available for over a decade. The BECT process, as implemented by .decimal (Sanford, FL), uses an external software (p.d) to design the patient‐specific bolus, which once designed is transferred to the clinic's treatment planning system as a structure for use in calculating the final dose plan. In parallel the bolus design is transmitted to .decimal for fabrication. The fabricated bolus is sent to the clinic, where its construction is typically verified by performing a dose calculation using a CT scan with the fabricated bolus in place. Other steps such as collimator fabrication, MU calculations are the same as for an electron beam therapy treatment without conformal bolus. This incremental process can be completed in a 2–3 day time frame.

IM‐BECT is a BECT treatment with the addition of an intensity modulator; however, there are significant changes to the process. First, IM‐BECT must be planned by an electron planning system that can design intensity modulators and compute dose for electron beams with intensity modulation, a feature unavailable in current treatment planning systems. Such a commercial system is currently under development. Because intensity modulation is designed in conjunction with bolus design, required planning time should have insignificant increase. Second, the intensity modulator requires fabrication, which is done in parallel with bolus fabrication, adding no increase to the pretreatment time. As the intensity modulator is rigidly embedded in the electron cutout, the cutout must be fabricated as part of the intensity modulator fabrication process. This is consistent with the concept that the cutout is an intensity modulator transmitting a relative electron fluence of unity inside and zero outside its aperture. Third, once received, QA of the intensity modulator and cutout can be performed using the methodology discussed earlier. This step could increase the time to treatment by a day. To clinically implement IM‐BECT, staff competence in electron beam therapy, understanding of the IM‐BECT process and its clinical utility, availability of an IM‐BECT planning system, and access to a 2D matrix detector like MapCHECK for intensity modulator QA are required competencies and equipment.

### 4.6 Failure mode and effects analysis (FMEA)

As IM‐BECT modulates range (energy) and intensity versus off‐axis position using physical boluses and intensity modulators, respectively, it provides a significantly advanced electron beam therapy technology, which is comparable to advanced x‐ray and proton therapies. Although QA procedures exist for BECT and additional ones are presented here for IM‐BECT, both modalities could benefit from a study of its workflow using FMEA analysis.^31^ FMEA analyzes have been reported for special electron techniques, such as total skin electron therapy^32^ and intraoperative electron therapy,^33^, ^34^ and the IM‐BECT workflow process reported here could benefit from such analysis once all steps are finalized and an initial commercial product becomes clinically available.

## 5 SUMMARY AND FUTURE DEVELOPMENT

This work describes and demonstrates for the first time techniques comprising a clinical process for intensity modulated bolus electron conformal therapy (IM‐BECT), which is an expansion of the process for bolus electron conformal therapy (BECT). It describes how (1) an intensity map operator is integrated into the BECT treatment planning process, (2) an intensity map is converted (segmented) into a set of island blocks that comprise the intensity modulator, (3) the PBRA dose algorithm is modified to compute dose in the presence of the intensity modulator, (4) the intensity modulator is fabricated, and (5) how quality assurance is performed at the factory and the clinic. This process was demonstrated for two patient examples, both previously treated using BECT, one a postmastectomy chest wall and one a temple.

The demonstration illustrates the feasibility for having an efficient, potentially economical method for delivering IM‐BECT using existing electron beam treatment machines. Software for planning IM‐BECT, which requires modifications to current clinically available BECT software (p.d) and interfacing it to other treatment planning systems, has been produced in a research capacity and is currently being further developed as part of an NIH SBIR grant for technology transfer. Also, factory fabrication of patient‐specific intensity modulators has been demonstrated, as have methods for factory and clinical quality assurance. As part of an NIH SBIR grant, improvements to the PBRA dose calculation, improvements to the factory quality assurance process, and methods for improving efficiency and setting thresholds for clinical quality assurance are being researched and developed. Also, as part of the grant and as this technology becomes available, its clinical utility will be studied, that is, which patient sites and characteristics might benefit most from IM‐BECT as compared to existing BECT and IMXT.

## CONFLICT OF INTERESTS

Kevin Erhart is an employee of .decimal, LLC and principal investigator of NIH Award Number R41CA199838 for which Mary Bird Perkins Cancer Center has a consortium agreement. The authors have no other relevant conflicts of interest to disclose.

## AUTHOR CONTRIBUTIONS

As submitting author, I attest that all coauthors and myself (1) contributed to drafting and/or editing the submitted manuscript, (2) reviewed and approved the final submitted manuscript, and (3) are accountable for the integrity of the material submitted. Each individual contributed in multiple ways to the material reported in this manuscript, and the primary contribution(s) from each author are:

- Elizabeth N. Hilliard was a graduate student who developed the IM‐BECT treatment plans, performed clinical QA measurements for the intensity modulators, and analyzed that data.
- Robert L. Carver supervised the graduate student and modified bolus design and PBRA dose algorithms used to design the intensity modulators.
- Erin L. Chambers developed and tested the algorithm for segmenting an intensity distribution into the matrix of pins that could provide such an intensity distribution.
- James A. Kavanaugh selected bolus ECT patients that would be suitable for demonstrating the utility of intensity modulated BECT(IM‐BECT) and provided their HIPPA compliant CT data, patient structures, and bolus plans for IM‐BECT planning.
- Kevin J. Erhart assisted with modifying software needed for the treatment planning, developed the factory construction process of the intensity modulators, and developed the factory QA process for the fabricated intensity modulators.
- Andrew S. McGuffey assisted with the writing of the manuscript as well as the development of the IM‐BECT treatment plans used in the manuscript.
- Kenneth R. Hogstrom conceived the concept of intensity modulated bolus electron conformal therapy (IM‐BECT), wrote the grant that funded the project, contributed to the data analysis, and supervised and coordinated efforts of all coauthors.
